# Dental Caries

**Published:** 1883-07

**Authors:** W. D. Miller

**Affiliations:** Berlin.


ARTICLE IV.

### DENTAL CARIES.—(CONTINUED.)

BY DR. W. D. MILLER, OF BERLIN.

There are various kinds of bacteria which have the power of forming a coloring matter, soluble in the medium in which they vegetate, so that not only the organisms themselves as seen *en masse*, but their substratum as well, acquire a deep red, blue, violet, yellow or other color. But in the thousands of sections of carious dentine which I have made I have never yet met with anything in any way entitled to be called a pigment bacterium, nor am I aware that any observer who has made an exhaustive study of the different species of micro-organisms found in the human mouth, has ascribed to them any power to produce pigment. Thin sections of very carious dentine show, even to the naked eye, the perfectly colorless patches of micro-organisms. Moreover, I have never seen any color in carious dentine which I have not repeatedly produced by the action of acids, or exposure to the air.

Specimens prepared as indicated under (*c*.), contain very often, not a trace of an organism. It requires a great amount of care to be sure that you have remove all the softened dentine and no more, as a layer 1–100 m. m thick, left in the bottom of the cavity, might be sufficient to destroy the value of the experiment. I use for this purpose a blunt spoon-shaped excavator.

Specimens prepared according to (*d*.), show that a limited number of organisms have entered the tubules, a fact of little importance, because, as above suggested, if we should fill a system of glass tubes of the same diameter as the dentinal tubes with organic matter, and place it for some months in a septic liquid, a section made parallel to and through the middle of one of the tubes would most likely reveal micro-organisms of some kind, but we would scarcely accuse them of an attempt upon the glass itself.

I suppose a coccus could enter a dead dentinal tubule about as readily as a glass tube of the same diameter; to ènter a living tubule he must, of course, first overcome the resistance offered by the dental fibril, which he undoubtedly can do. This does not signify that he can in any way attack the unchanged basis substance. As a matter of fact the sections under consideration show no anatomical changes whatever; no expansion of the tubules, no breaking through of the basis substance, or any other structural change. Examined microscopically they do not appear to be changed in the least, the edges and corners being as sharp and hard as when they were put in the flasks nine months before, although I have taken care, by frequently adding fresh carious dentine, that the state of the liquid should be as septic as anything ever found in the human mouth, and much more so, and have kept them at an agreeable temperature (cold, hard dentine doesn't seem to be to their taste), yet they seem to have made no impression upon the pieces used.

Again, if we examine a freshly extracted carious tooth, we will find, say on the grinding surface the enamel broken through and the dentine softened to the depth of 1 to 2 m. m. Now let us suppose that this whole process has been caused by fungi alone. If, then, we place the tooth under conditions of moisture and temperature favorable to the development of these fungi, the carious process should continue. The enamel should dissolve away more and more, and the dentine become softened deeper and deeper. This experiment I have repeatedly tried, and have invariably found that after a lapse of from three to five weeks, the dentine which was previously softened has become entirely consumed. The softening has, however, entirely ceased, and the walls are found to be perfectly hard, and if the cavity is syringed out with water it will be found to be as clean as though some scrupulous dentist had prepared it for filling. In other words, we obtain a result entirely contrary to our expectations and as unlike ordinary caries as it could well be.

I have made but four microscopic preparations from such teeth. Three of them do not contain a single coccus which may not be called accidental, while the other shows half a dozen tubules containing as many cocci as might be counted on one's fingers, the tooth not having remained sufficiently long in the liquid for all the softened dentine to be consumed.

It appears to me, on the whole, that the idea that any Tom, Dick or Harry of the world of fungi, who happens to get into the human mouth, at once sets to work to pick the teeth to pieces, is without much show of reason, and to make caries absolutely dependent upon these same agents is still more questionable.

Preparations of the kind indicated under (*d*), are sometimes very instructive. I will describe only one case. A section was ground from a tooth which had been over thirty years in the human mouth. The section revealed a circle of interglobular spaces, extending completely around the pulp cavity just beneath the enamal. Through a deep, broad fissure in the enamel, cocci had gained access to the interglobular spaces, and, passing from one to the other, had filled every space for full three quarters of the distance around the section. Whether they had made that their home for one, three or twenty years, it is not possible to say. At any rate the dentine did not appear to have suffered in the least from their presence and only now and then a few cocci appeared to have forced an entrance into the dentinal tubuli for a fraction of a millimeter, without in any way encroaching upon or invading the basis substance.

I have said nothing as yet about enamel, which, whatever may be the cause of caries, is the first and strongest barrier to be overcome. I have a large number of preparations of carious teeth, comprising both the enamel and dentine, and I can only say that as far as I am at present able to judge, indications of a purely parasitical caries of the enamel are very meagre indeed.

To sum up my views of the etiology of *caries dentium* then, I believe as follows:

1st. Caries of the teeth is not entirely of a parasitic nature.

2d. The first stage of dental caries consists in a softening of the tissue of the tooth.

3d. In effecting the softening, acids, particularly those generated by fermentation, often play a very important part.

4th. Whether micro-organisms are or are not concerned in the production of the first stage of caries, or whether any other agent is concerned in the process, (be it in the form of a simple solvent, or be it a pathological action on the part of the tooth itself), are questions which certainly, as yet, cannot be satisfactorily answered.

5th. Destruction of the enamel may take place quite independently of micro-organisms.

6th. The second stage of caries, devitalization and breaking down of the softened tissues, is entirely parasitic, and is inaugurated chiefly, if not wholly, by the elements of the Leptothrix buccalis, while later on various other fungi may take part in the decomposing process.

From this it may be seen that I am a believer in acids, a believer in micro-organisms, and a believer in an unknown cause; that is, I believe that there are agent active in the producing of caries which are yet to be discovered.

I have attempted in this hurriedly-written article, not so much to point out what the fungi of caries can do, as what they do not do, or rather what my preparations do not prove that they do, for although their powers for evil are very great, yet I believe that there is a tendency to overestimate them, and to jump to conclusions which can hardly be said to be sufficiently well supported by facts.

